# Graphene Nanopore Fabrication and Applications

**DOI:** 10.3390/ijms26041709

**Published:** 2025-02-17

**Authors:** Qijiao Sun, Min Dai, Junjie Hong, Silu Feng, Chengyong Wang, Zhishan Yuan

**Affiliations:** 1 School of Electromechanical Engineering, Guangdong University of Technology, Guangzhou 510006, China; sqj1234567892021@163.com (Q.S.); dm332000@163.com (M.D.); cywang@gdut.edu.cn (C.W.); 2 Guangdong Provincial Key Laboratory of Minimally Invasive Surgical Instruments and Manufacturing Technology, Guangdong University of Technology, Guangzhou 510006, China; 3 State Key Laboratory for High Performance Tools, Guangdong University of Technology, Guangzhou 510006, China; 4 Smart Medical Innovation Technology Center, Guangdong University of Technology, Guangzhou 510006, China; 5 School of Integrated Circuit, Guangdong University of Technology, Guangzhou 510006, China; 2112425034@mail2.gdut.edu.cn

**Keywords:** graphene nanopores, fabrication technology, biomolecule detection, molecular separation, osmotic power generation

## Abstract

Graphene is a revolutionary material with excellent optical, electrical and mechanical properties and has garnered significant attention in the realm of nanopore technology. Devices incorporating graphene nanopores leverage the material’s atomic thickness to enhance detection precision in solid-state nanopores. These nanopores exhibit high spatial resolution and ion selectivity, making them promising sensors for biomolecular detection. Additionally, their unique characteristics suggest their considerable potential for applications in material separation and osmotic power generation. In recent years, several literature reviews on graphene nanopores have been published; however, some have not fully addressed certain important aspects, such as the depth of theoretical analysis, the extent of coverage on technological advancements, and the exploration of potential applications. This paper reviews current fabrication methods, including “top-down” etching and “bottom-up” synthesis, highlighting their advantages and limitations. We also summarize diverse applications of graphene nanopores, such as in biomolecule detection and water desalination. Our findings emphasize the need for a deeper exploration of these aspects, advancing the field by showcasing the broader potential of graphene nanopores in addressing various technological challenges.

## 1. Introduction

Over the past few decades, the development of single-nanopore sensors from various materials has yielded highly sensitive platforms for biomolecular detection, with a particular focus on signal molecules. These nanopore sensors are generally classified into two major categories: biological nanopores and solid-state nanopores. A seminal study by Kasianowicz et al. [1] demonstrated that an individual nucleobase (α-hemolysin, α-HL) can be identified on a nanopore’s static strand. Since then, nanopore technology has advanced significantly, with nanopore fabrication [2,3] and application [4,5,6] due to their tunable pore size, chemical stability, and potential for integration into electronic systems. Biological nanopores are normally introduced into a membrane composed of liposomes, lipid bilayers, or polymers. This type of nanopore is highly reproducible in specific size and structure and has been used in established genetic techniques such as site-directed mutagenesis or the incorporation of specific adaptors. Bio-nanopores show great potential as fast, label-free, real-time, low-cost, and easy-to-modify materials [7]. They provide excellent reproducibility and high sensitivity for single-molecule analysis. Various bio-nanopores such as α-HL [8], MspA [9], phi29 [10], OmpG [11], and ClyA [12], as well as dual-constriction biological nanopores [13], have been used in the detection of DNA [14,15], RNA [16,17,18], protein [19,20], metal ions and small organic molecules [21]. Notably, bio-nanopores have successfully achieved DNA sequencing [22,23].

Solid-state nanopore detection technology represents a powerful approach for addressing key challenges in molecular detection, such as enhancing sensitivity at the single-molecule level, achieving precise target detection, and optimizing performance in terms of anti-interference capabilities and high-throughput, real-time analysis. Significant advancements have been made in the development of both qualitative and quantitative analytical methods for target molecules using this platform. The ability to analyze molecular structure and function at the single-molecule level, coupled with real-time biological analysis, constitutes a critical area of research in nanopore sensor development. Currently, solid-state nanopores have been developed using various materials, including silicon nitride (Si_3_N_4_) [24], silicon oxide (SiO_2_) [25], alumina oxide (Al_2_O_3_) [26], zinc oxide (ZnO) [27], hafnium oxide (HfO_2_) [28] and glass [29]. These nanopores have been employed in numerous applications, such as detecting DNA and RNA conformations [30,31,32], discriminate between DNA homopolymers [33] and analyzing protein primary structure [34]. Despite advancements enabling the creation of nanopores smaller than 1 nm in diameter [35], solid-state nanopores have yet to successfully accomplish DNA sequencing. Additionally, solid-state nanopores are capable of distinguishing between monomer and aggregate proteins [36,37], as well as detecting various virus types [38,39]. However, their utility is constrained by limitations in spatial resolution [3,7,40]. To address this issue, the use of thinner membranes has been identified as a strategy to enhance spatial resolution [34,41,42]. Particularly, two-dimensional materials, such as graphene [43], boron nitride (BN) [44], molybdenum disulfide (MoS_2_) [45] and tungsten disulfide (WS_2_) [46], have been explored for fabricating solid-state nanopores. Among these two-dimensional materials, graphene is the thinnest of these materials, with a single layer measuring only 0.335 nm [47], closely matching the interbase distance (~0.3 nm) found in DNA molecules. This atomic thickness significantly improves spatial resolution in single-molecule detection. In addition to its atomic thickness, graphene possesses several unique properties, including high flexibility, an excellent ion-selective ability, and superior thermal and electrical conductivity [48,49]. These characteristics have garnered considerable attention for graphene nanopores in diverse applications, such as biomolecule detection [50], seawater desalination [51], molecular separation [52] and osmotic power generation [53] applications.

Although several literature reviews on graphene nanopores have been published in recent years, many of these studies appear to have certain limitations. First, some reviews lack sufficient depth in theoretical analysis, with key aspects such as the formation mechanisms and electronic transport properties of graphene nanopores not being explored in great detail, which may result in an incomplete understanding of these fundamental topics. Second, the coverage of technological advancements is sometimes not fully up to date, as many reviews do not fully capture the latest developments in fabrication techniques and characterization methods, potentially overlooking recent innovations. Third, the exploration of potential application areas tends to be somewhat narrow, with many reviews focusing on a limited set of applications, thereby not fully addressing the broader possibilities of graphene nanopores in fields like biosensing and energy storage.

To address these gaps in the literature, this mini review presents an overview of graphene nanopore fabrication methods. It further emphasizes the applications of graphene nanopores and nanopore arrays over the past decade. Specifically, the prominent applications of graphene nanopores in biomolecule detection, water desalination, gas separation, heavy metal ion separation, and osmotic power generation are discussed in detail.

## 2. Graphene Nanopore Fabrication Method

### 2.1. Suspended Graphene Film Preparation

Graphene nanopore fabrication typically comprises three fundamental steps: substrate preparation, graphene transfer and nanopore creation [43]. The substrates used are generally silicon chips coated with silicon nitride or silicon oxide. Photolithography and reactive ion etching (RIE) are employed to define a KOH etching window on the substrate. Subsequent KOH etching results in the formation of a suspended nano membrane. Miro-or nanopores are then fabricated in this suspended nanomembrane using RIE or focused ion beam (FIB) methods. Finally, transferring the graphene sheet onto the substrate yields a suspended graphene layer.

Graphene sheets are currently prepared using various methods, including mechanical exfoliation [48], chemical exfoliation [54], epitaxial growth [55], oxidation–reduction [56], and chemical vapor deposition (CVD) [57]. Mechanical exfoliation, the earliest technique employed to obtain single-layer graphene sheets, offers high-quality graphene but requires advanced equipment. While chemical exfoliation and oxidation–reduction methods enable large-scale production at lower costs, their poor controllability and quality limit their applications in the nanopore area. In contrast, graphene fabricated by CVD method exhibits uniform thickness over large areas, with successful demonstrations of monolayer 30-inch graphene films [58]. This mass production method for graphene functional devices is compatible with MEMS technology, thereby expanding graphene’s range of applications. Consequently, most graphene nanopores have been fabricated using either mechanical exfoliation [59] or CVD [60] methods, as these techniques allow for precise control over graphene thickness. Additionally, the high quality and scalability of these methods enhance the reliability of the fabrication processes.

Subsequently, prior to graphene nanopore fabrication, the graphene film can be transferred onto the substrate surface [43,61]. Initially, polymethyl methacrylate (PMMA), commonly used as a supporting layer during the transfer process, is applied to the graphene surface. The growth substrate of CVD graphene or the substrate of mechanical exfoliation is then removed through methods such as dissolution in a liquid etchant, bubble delamination, or thermal peel-off [62,63], resulting in the acquisition of graphene/PMMA film. This graphene/PMMA film is then placed onto the substrate designated for graphene nanopore fabrication. Finally, the PMMA layer is dissolved using organic solvents like acetone. After washing and drying, the exposed graphene is suspended over the substrate surface. Graphene nanopores are subsequently fabricated on suspended graphene using various techniques, which will be discussed in detail in the subsequent section.

### 2.2. Fabrication Methods of Graphene Nanopores

**Figure 1:**
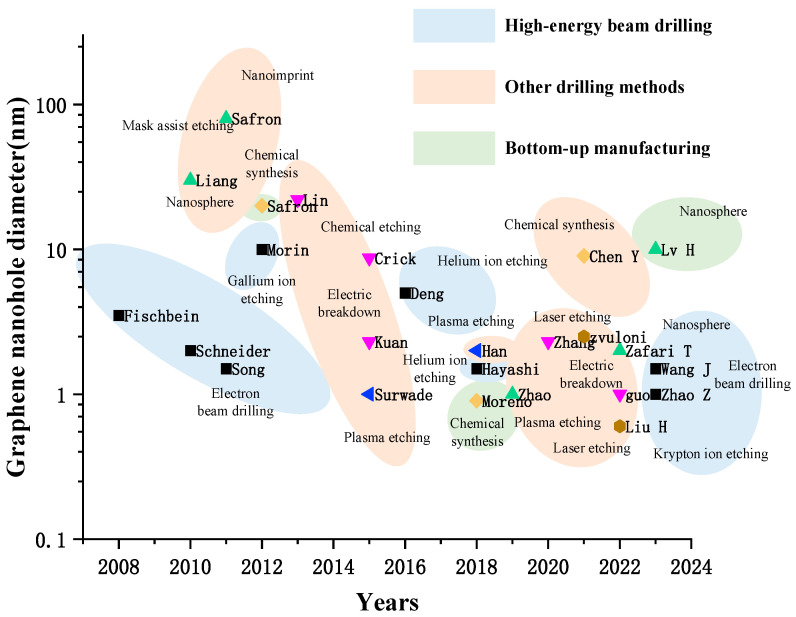
The development of graphene nanopore fabrication methods with accuracy [57,60,61,64,65,66,67,68,69,70,71,72,73,74,75,76,77,78,79,80,81,82,83,84,85].

The main fabrication methods for graphene nanopores include electron beam milling, ion beam etching, plasma etching, electric breakdown, chemical etching, chemical synthesis, photothermal effect etching and laser etching. In this section, we provide a detailed introduction to the characteristics of each fabrication technology. Additionally, we discuss their respective advantages and disadvantages. Figure 1 presents a comprehensive summary of the development of graphene nanopore fabrication methods, highlighting their precision and accuracy.

#### 2.2.1. Electron Beam Sculpting

**Figure 2:**
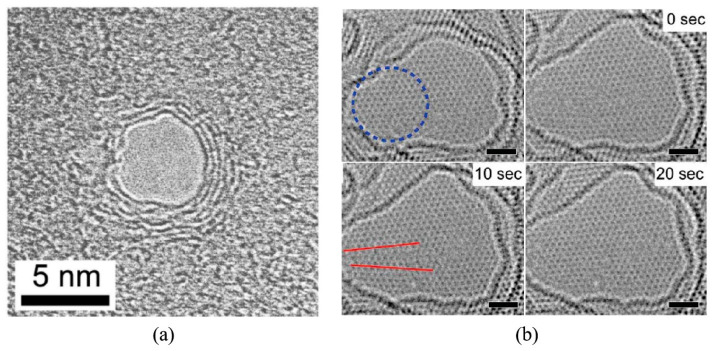
(**a**) TEM imaging of graphene nanopores [59]. (**b**) High resolution electron microscope (HREM) images illustrating the self-repair occurring in a graphene monolayer at 600 °C; the graphene subsequently recovers its single crystallinity with ~20 s [87], The blue dashed circle is positioned at 80% of the maximum beam intensity. Between the two red lines, the regular lattice structure has undergone amorphization due to intense electron beam irradiation. Scale bars 1 nm.

Graphene nanopores with diameters as small as 3.5 nm have been successfully fabricated using FIB irradiation for 5 s in transmission electron microscope (TEM) [86]. This method also enables the realization of graphene slits and gaps, demonstrating that graphene nanostructures can be effectively processed at the nanoscale using electron beam irradiation. Subsequently, Schneider et al. [59] produced graphene nanopores with diameters ranging from 2 to 40 nm by employing a focused electron beam at an acceleration voltage of 300 kV. However, the researchers found that focused electron beam irradiation in TEM can introduce undesired defects and artifacts, such as fast amorphization of the graphene crystal structure and carbon deposition, which may adversely affect the performance of the graphene nanopores. Fortunately, Song et al. [87] identified a significant phenomenon: the self-repair of graphene occurs at high temperature (>600 °C) in TEM, as shown in Figure 2. The self-repair phenomenon of graphene nanopores significantly influences the structural mechanism of nanopores through the migration and filling of vacancy defects by carbon ad-atoms at high temperatures (>600 °C). Under electron beam irradiation, carbon atoms are ejected from the graphene lattice, creating vacancy defects. However, at elevated temperatures, carbon ad-atoms rapidly migrate and fill these vacancies, repairing the lattice and preventing amorphization. This repair mechanism ensures atomic-level precision and crystalline integrity at the nanopore edges, maintaining a single-crystalline structure during nanopore formation. Furthermore, carbon ad-atoms not only fill vacancies but also form new C-C bonds with carbon atoms at the nanopore edges, enhancing edge stability and enabling the nanopores to retain their geometric shape and size after formation, thereby preventing further damage or deformation. The self-repair mechanism also allows for precise control over the size and shape of nanopores during the sculpting process. By adjusting the intensity and focus of the electron beam, carbon atoms are gradually removed to form the desired nanopore structures, ensuring atomic-level smoothness and regularity at the edges and achieving high-precision fabrication. Additionally, the self-repair mechanism is equally effective in multilayer graphene. Each layer maintains its crystalline structure through the migration and filling of carbon ad-atoms, ensuring the stability and consistency of nanopores in multilayer graphene and preventing the disruption of individual layers from affecting the overall structure. In summary, the self-repair phenomenon, through the migration and filling of vacant defects by carbon ad-atoms, preserves the crystalline structure of graphene and the stability of nanopores. It ensures atomic-level precision and crystalline integrity at the nanopore edges, enables high-precision control over the size and shape of nanopores, and is applicable to both single-layer and multilayer graphene structures. This self-healing phenomenon has been shown to mitigate the deficiencies introduced by focused electron beam irradiation. Moreover, high-precision images provided by TEM in real time during nanopore sculpting enhance the fabrication process [3,64]. Therefore, the electron beam in TEM has been considered feedback control technology used for fabricating graphene nanopores with extremely high manufacturing accuracy and quality. However, due to the limitations of the TEM sample holder, only one substrate can be loaded into the high vacuum chamber at a time, significantly reducing the manufacturing efficiency of graphene nanopores. Specifically, the high vacuum environment of the TEM requires that the sample undergo complex vacuum pumping and venting procedures during loading and unloading to ensure that the vacuum integrity of the chamber is not compromised. This process is not only time-consuming but also limits the ability to process multiple samples simultaneously. Furthermore, after each loading and unloading of the sample, the system requires the recalibration of the electron beam alignment and focus to ensure the precision of the sculpting process, which further increases the manufacturing time. Due to these technical constraints, the fabrication process of graphene nanopores becomes extremely slow, making it unsuitable for large-scale or high-throughput production.

The scanning electron microscope (SEM) has been investigated as a new tool to enhance the manufacturing efficiency of graphene nanopores. Sub-10 nm graphene nanopores have been achieved using N_2_-assisted low-energy (<10 keV) focused electron beams [65]. Specifically, when a low-energy (<10 keV) electron beam in an SEM irradiates the graphene surface, the beam interacts with nitrogen gas molecules, causing their ionization and generating highly chemically reactive nitrogen ions (N₂⁺). These nitrogen ions then react with carbon atoms on the graphene surface, forming gaseous products such as cyanogen (CN)_2_. These gaseous products detach from the graphene surface and are evacuated through the SEM chamber’s vacuum system, leading to the localized removal of carbon atoms from the graphene surface and the creation of vacancy defects. Once an initial small pore is formed on the graphene surface, the edges of the pore become preferential sites for further reactions with nitrogen ions, which continue to react with carbon atoms at the pore edges, causing the pore to expand radially. Due to the diffusion of nitrogen ions, the etching effect extends beyond the region directly irradiated by the electron beam, forming nanopores. The etching rate is influenced by several factors, including nitrogen gas pressure, electron beam current, and electron beam energy. Higher nitrogen pressure and electron beam current increase the generation of nitrogen ions, thereby accelerating the etching rate, while lower electron beam energy also increases the etching rate, as low-energy electrons interact more strongly with nitrogen gas and graphene, leading to more nitrogen ion generation and higher energy transfer efficiency. During the etching process, the graphene lattice undergoes structural disruption and amorphization; the removal of carbon atoms creates vacant defects in the lattice, and surrounding carbon atoms rearrange due to surface tension, further exacerbating lattice damage. Ultimately, these damaged regions expand and form nanopores. This principle is also applicable to low-energy (5 keV) electron beams [88], where graphene nanopores with diameters as small as 5 nm are etched under low-pressure (0.3 Pa) nitrogen. The etching rate can be controlled by the pressure of the etching gas, the electron beam current, and the electron beam energy. In addition to nitrogen ions, water vapor and ionizing oxygen ions [66] have also been demonstrated to facilitate the fabrication of graphene nanostructures with the assistance of electron beams. This new technology enriches the methods available for graphene nanopore manufacturing. Although the machining precision of graphene nanopores using electron beams in TEM is higher than that in SEMs, the aforementioned improvements in using SEMs provide a new strategy to compete with TEM. Furthermore, the advantage of the large sample holder in an SEM enhances its application prospects in future research.

#### 2.2.2. Focused Ion Beam Etching

**Figure 3:**
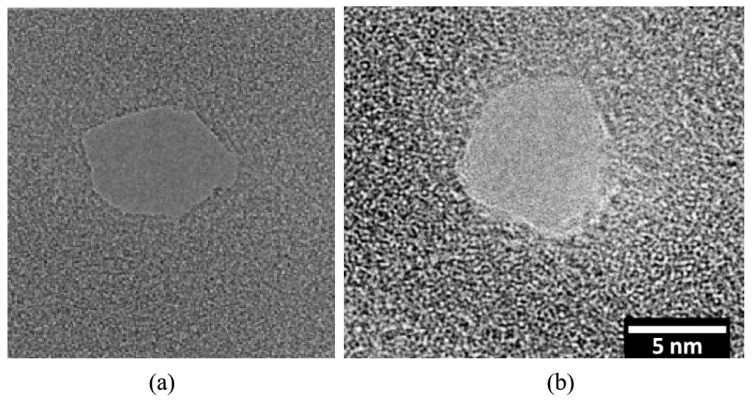
TEM image of graphene nanopores. (**a**) Gallium ion beam manufacturing graphene nanopores [90]. (**b**) HIM manufacturing graphene nanopores [89].

FIB etching is a high-energy technique utilized for the fabrication of graphene nanopores. When high-energy ions collide with carbon atoms in graphene, sputtering occurs, leading to the formation of graphene nanopores [89]. The most commonly used ion sources for graphene nanopore etching are gallium and helium ion beams. Morin et al. [90] employed Ga^+^-source FIB technology to create irregular nanopores with diameters of 10 nm in graphene membranes, as shown in Figure 3a. The irregular morphology of these graphene nanopores is attributed to residual stress within the suspended graphene arising during the transfer process. Recent advancements in FIB technology have enabled the successful fabrication of circular graphene nanopores with a beam exposure time of 0.1 s [91]. This fabrication approach demonstrates significant advantages over traditional TEM-based electron beam sculpting methods, particularly in terms of throughput capability, as it allows for the simultaneous processing of multiple chips. However, technical limitations have been identified regarding the precision of gallium ion (Ga^+^)-based FIB systems, which currently cannot achieve the direct fabrication of graphene nanopores with diameters below 10 nm. This resolution highlights the need for further development in ion beam technology to enable sub-10 nm feature fabrication in two-dimensional materials.

Unlike the Ga^+^-source FIB system, He^+^-source systems offer a significantly smaller beam spot size (<0.5 nm), and the mass of He^+^ is also smaller than that of Ga^+^. These characteristics enhance the manufacturing resolution and quality of graphene nanopores, enabling almost damage-free fabrication. The fabrication of graphene nanopores with precise dimensions was achieved through helium ion microscope (HIM) irradiation, as illustrated in Figure 3b. Experimental observations revealed a non-linear relationship between nanopore diameter expansion and ion beam irradiation duration [89]. Specifically, the initial stage of irradiation demonstrated accelerated pore growth, followed by a gradual reduction in expansion rate during prolonged exposure. This characteristic growth pattern was mechanistically explained by the Gaussian distribution profile of the He^+^ beam intensity, where the central region exhibited higher ion density compared to the peripheral areas. The etching kinetics of graphene were found to be directly proportional to the local helium ion intensity, resulting in progressively slower nanopore enlargement as the etching process continued. These findings provide crucial insights into the time-dependent etching behavior of graphene under focused ion beam irradiation, which is essential for achieving precise control over nanopore dimensions in nano-fabrication processes. Following this work, Hayashi et al. [67] fabricated graphene nanopores with a diameter of only 1.5 nm by HIM. The formation of nanopore can be well reproduced by controlling the intensity of the ion beam. Then, Schmidt et al. [68] created graphene nanopore arrays with a 10 nm pitch and 4 nm diameter using helium ion beams in the same year. In summary, the manufacturing accuracy of HIM is comparable to that of electron beams in TEM. Meanwhile, the large-scale manufacturing capabilities of HIM [68,69] demonstrate that He^+^ beam etching may be one of the most promising methods for graphene nanopore arrays at the wafer scale in the future.

#### 2.2.3. Plasma Etching

**Figure 4:**
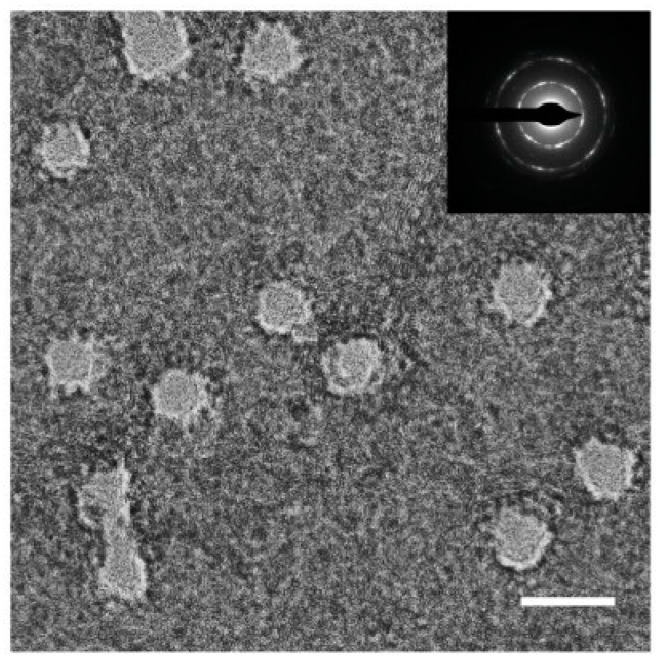
Scanning transmission electron microscope (STEM) images of graphene nanopores after exposure to O_2_ plasma [92]. Scale bars is 10 nm.

High-energy beam sculpting methods effectively control the size and shape of graphene nanopores; however, the high cost of the equipment limits their widespread application. To address this issue, researchers have sought alternative, more economical methods for the fabrication of graphene nanopores. Plasma etching, a commonly used technique, generates defects in graphene by etching carbon atoms from the lattice, resulting in the formation of nanopores in the vacancy defect regime [70]. Zandiatashbar et al. [70] found that defects appeared in the graphene membrane following continuous oxygen plasma etching. Despite a decline in the mechanical properties of the graphene, the membrane remained suspended independently without breaking or collapsing. These results suggest that plasma etching may serve as a viable method for creating graphene nanopores. Subsequently, a large-area array of graphene nanopores (5 × 5 cm) was fabricated using O_2_ plasma etching [71]. However, the distribution of nanopore diameters varied significantly, ranging from 200 nm to 900 nm. During O_2_ plasma etching, both the diameter and the number of nanopores were controlled by the etching time [92]. When the etching time was reduced to 1.5 s, graphene nanopores with a diameter of 1 nm were fabricated, as shown in Figure 4. In 2018, Han Qi et al. [93] used O_2_ and Ar as plasma gasses for etching graphene. Interestingly, extending the O_2_ plasma etching time resulted in an increase in both defect density and defect size, whereas prolonged argon plasma etching only enhanced defect density. A high density of graphene nanopores (2.1 × 10^12^ cm^−2^) was also achieved through O_2_ plasma etching [94]. This high density of graphene nanopores has proven effective for applications in gas mixture separation. Additionally, high-density graphene nanopores can also be fabricated using a two-step plasma etching process [95]. In this method, the diameter and surface charge density of the graphene nanopores are controlled through a combination of argon and oxygen plasma or hydrogen plasma method. Initially, the graphene membrane is treated with argon plasma, followed by treatment with either oxygen plasma or hydrogen plasma. Beyond controlling pore size, the surface charges of the nanopores can be regulated by the different functional groups at the pore rims, which are formed by oxygen or hydrogen plasma. The resulting nanoporous graphene membranes exhibit the selective filtration of cations with different hydration radii and can effectively distinguish between cations and anions, achieving high selectivity. This capability is attributed to the synergic effects of nanopore size and surface charge.

**Figure 5:**
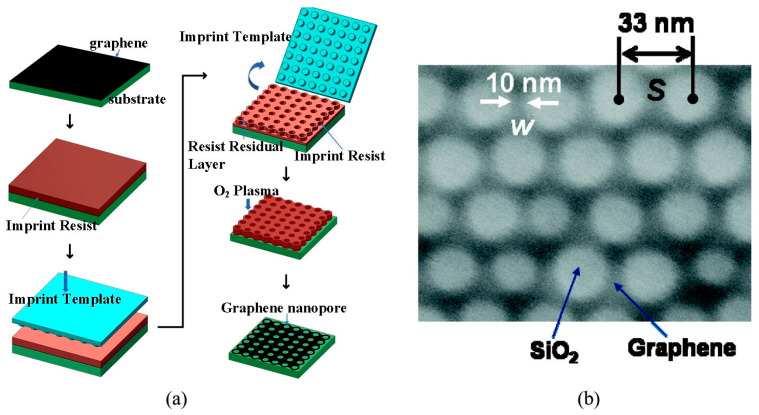
Mask-assisted etching process [96]. (**a**) Nanoimprinting manufacturing process. (**b**) SEM image of graphene nanopores.

Plasma etching represents a cost-effective and straightforward approach for the large-scale production of graphene nanopores. However, it suffers from the poor location and size controllability of the nanopores. To enhance the controllability of plasma etching, template-assisted etching has been used to define the specific areas where a large number of graphene nanopores are generated. Initially, a porous template or mask is placed on the surface of the graphene. Then, plasma etching is applied to etch graphene beneath the template. Finally, graphene nanopores are obtained after the removal of the porous template or mask. Common materials used as porous templates or masks include block copolymers, nanospheres, nanoporous alumina templates, and patterned photoresist layers. Moreover, nanoimprint technology [96] has been used to create patterns, resulting in a graphene nanopore array with diameters of approximately 30 nm, as illustrated in Figure 5a. Nanospheres serve as another template material, where the diameter of the nanopores can be controlled by the size of the nanospheres. Safron et al. [72] used 72 nm and 290 nm polystyrene nanospheres as templates to fabricate high-density graphene nanostructures. Furthermore, pristine graphene has poor hydrogen storage characteristics, and addition of dopants like boron and nitrogen or decoration by transition metals significantly improves the performance. In addition, graphene allows the tuning of surface curvature which can help in achieving a reversible hydrogen storage system with fast kinetics [73].

In summary, plasma etching, particularly O_2_ plasma etching, has become an effective and straightforward method for the fabrication of graphene nanopores. The controllability provided by template-assisted etching facilitates the rapid production of large-area graphene nanopores with periodic arrays. Furthermore, the low cost and high manufacturing efficiency of plasma etching make it increasingly viable for practical applications.

#### 2.2.4. Electric Breakdown

**Figure 6:**
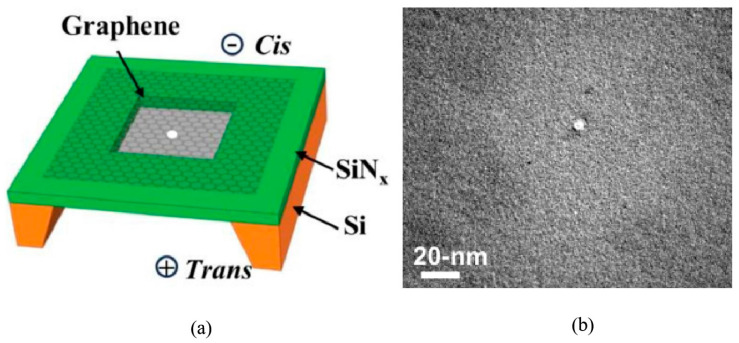
Dielectric breakdown method. (**a**) Principle of dielectric breakdown method [99]. (**b**) TEM image of electrical pulse-fabricated pore [74].

The electric breakdown method is a simple and cost-effective technique for fabricating graphene nanopores through the repeated application of short high-voltage pulses on both sides of the graphene membrane. This technology has successfully produced solid-state nanopores with diameters less than 5 nm [74], as shown in Figure 6a. In this method, the diameter of nanopores is indirectly controlled by monitoring the ionic current. The capability for size control was first validated in the fabrication of Si_3_N_4_ nanopores [75,97,98]. Like the Si_3_N_4_ nanopore fabrication process, fluctuation in leakage current is monitored to determine whether a graphene nanopore has been generated during the application of pulse voltage to both sides of the graphene membrane [99], as shown in Figure 6b. Once a nanopore is generated, a brief pulse of low voltage is applied to expand the nanopore. According to the ionic current calculation formula, the pulse voltage is terminated in real time once the diameter of the graphene nanopores reaches the desired size, ultimately achieving sub-1 nm graphene nanopores. It is believed that the electrochemical reaction between graphene and electrolyte induces the removal of carbon atoms from the lattice [100]. Crick et al. [100] combined DNA translocation studies with pore-opening experiments, monitoring alterations in DNA translocation behaviors through the electrical breakdown process. This experiment suggests the potential to adjust nanopore diameters in real time based on the size of the analytes. Following this work, Zhang et al. [76] employed the electric breakdown method to create graphene nanopores on a glass substrate. In comparison to traditional Si_3_N_4_ substrates, the noise levels associated with graphene nanopores on glass substrates were significantly reduced.

The electrical breakdown method is a straightforward and economical approach for the high-precision fabrication of graphene nanopores. This method effectively controls nanopore size through ion current feedback. However, distinguishing between the enlargement of a single nanopore and the simultaneous formation of multiple nanopores remains a challenge as the ion current increases.

#### 2.2.5. Chemical Etching

**Figure 7:**
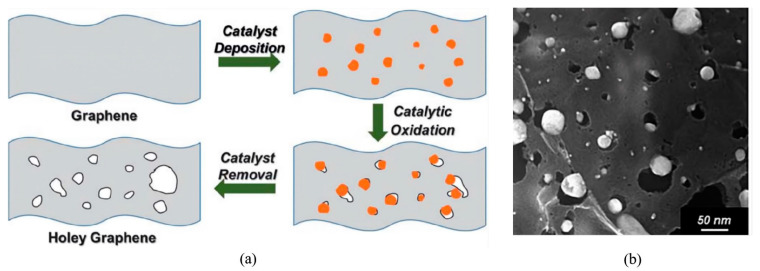
Fabricated graphene nanopores by catalytic oxidation [77]. (**a**) Thermally exfoliated graphene process. (**b**) SEM maps of nanopores prepared by chemical etching of silver particles.

Chemical etching serves as a simple and cost-effective method for fabricating graphene nanopores. This technique typically employs metals, metal oxides, or other chemical reagents to oxidate carbon atoms within the graphene membrane. The oxidation process generates carbon vacancies, resulting in the formation of nanopores, while gaseous products, such as CO or CO_2_, are produced during the nanopore formation process. Silver (Ag) nanoparticles have been shown to catalyze the oxidation of carbon in graphene at 300 °C [77]. As a result, nanopores with diameters ranging from 1 to 50 nm were created in the graphene membrane, as shown in Figure 7. X-ray photoelectron spectroscopy (XPS) characterization confirmed the catalytic role of Ag nanoparticles, which maintained a zero-valent state after the reaction. This suggests that the size of graphene nanopores can be tuned by varying the size of the Ag nanoparticles, as shown in Figure 7.

**Figure 8:**
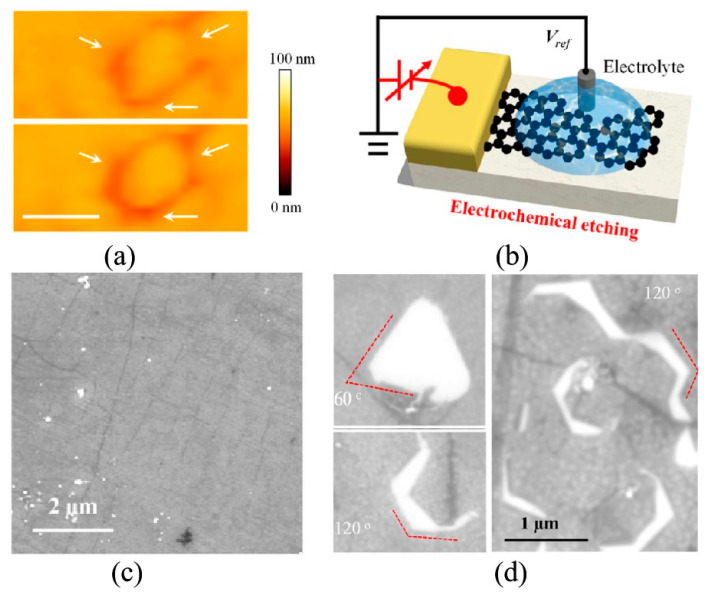
GOG nanopore formation via electrochemical etching [76]. (**a**) In situ liquid AFM images scanned at the same position during graphene nanostructure enlargement under electric pulses, as indicated by the white arrows (**upper panel**: before the pulses; **lower panel**: after the pulses). (**b**) A scheme of electrochemical etching of CVD graphene transferred on Al_2_O_3_(20 nm)/SiO_2_(285 nm)/Si substrate and the corresponding circuit diagram. (**c**) An SEM image of as-transferred CVD graphene on the Al_2_O_3_/SiO_2_/Si substrate. (**d**) SEM images of the CVD graphene after 30 min of 1.6 V electrochemical etching in 1 M KCl solution. The exposed hexagonal edges of the CVD graphene (on the insulation of the Al_2_O_3_/SiO_2_/Si substrate in white color) suggest that the electrochemical etching process is most likely initiated from its grain boundaries containing active defects.

Subsequently, a carbothermal reaction involving oxometallates (OMs) or polyoxometalates (POMs) and graphene was proposed to fabricate sub-10 nm nanopores [101]. In this reaction, carbon atoms in graphene serve as reducing agents, facilitating the decomposition and reduction of OMs and POMs into metal oxides, metals, and/or even metal carbide compounds, with carbon being oxidized as CO and CO_2_. The size of graphene nanopores can be controlled by adjusting the quantity of OMs or POMs in the precursor. Additionally, the reaction between graphene and oxygen in hot air (~500 °C) has been shown to produce nanopores [102], as seen in Figure 8. In this method, nanopore formation occurs at sites of non-crystalline spots (point or vacancy defects) where graphene initially reacts with oxygen. Factors such as the heating rate, temperature, and duration significantly influence the size, morphology, and density of the resulting pores in graphene. Furthermore, a hydrothermal reaction involving an aqueous solution of H_2_O_2_ and well-dispersed graphene oxide (aqueous) has been confirmed to generate a holey graphene framework [103]. However, prolonged heating at around 180 °C may lead to reduced manufacturing efficiency.

Electrochemical etching employs electrochemical reactions to remove material from surfaces, with the workpiece functioning as the anode and a corrosion-resistant metal serving as the cathode. When an electric current flows through the electrodes and electrolyte solution, it triggers reactions that dissolve and eliminate the targeted metal, ultimately leading to corrosion. In the nascent stages of nanopore fabrication, a glass chip is utilized as the supportive substrate for graphene, distinguished by its low capacitance and featuring a micrometer-sized aperture at its core. During the preparatory phase, an electrical pulse voltage is applied to the graphene layer deposited on the glass substrate. This voltage induces the removal of carbon atoms under the influence of the electric field, thereby facilitating the formation of nanopores within the graphene structure. This electrochemical etching technique not only facilitates the efficient creation of nanopores but also yields nanopores with exceptionally clean characteristics. Moreover, given that this methodology can be executed in a liquid environment, researchers have the capability to utilize in situ liquid atomic force microscopy (AFM) to delve into the electrochemical mechanisms involved, thereby fostering a deeper understanding of the nanopore formation process [99].

In summary, the chemical etching method offers an effective pathway for the large-scale and cost-effective preparation of graphene nanopores. However, the limitation of this technique lies in the relatively limited control over the diameter of the nanopores, and due to the inhomogeneity of the chemical reactions, the morphology of the graphene nanopores may exhibit certain variations [104]. Additionally, the etchants used in the chemical etching process may lack environmental friendliness, thereby raising environmental concerns.

#### 2.2.6. Bottom-Up Method

The bottom-up manufacturing of graphene nanopores involves the direct formation of these structures during graphene growth. This technology operates on the principle of constraining the graphene pattern to facilitate nanopore development during the growth phase. Two primary strategies are employed to achieve graphene nanopores.

**Figure 9:**
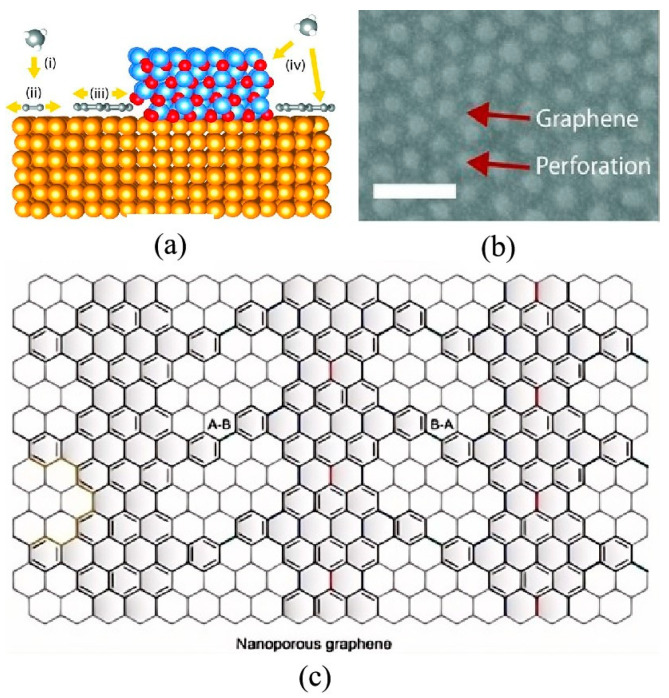
Graphene nanopores hindered by the deposition method. (**a**) Technological process [105]. Schematic of BG-CVD: (i) methane decomposes into C, which (ii) diffuses and nucleates graphene, (iii) growing until (iv) the entire Cu surface is covered. (**b**) TEM image of graphene nanopores [105]. (**c**) The A-B or B-A bounding combinations give rise to identical pores with different orientations [106].

The first is barrier-guided chemical vapor deposition (BG-CVD) [105]. In this method, the catalytic activity of the metal substrate is passivated to limit the generation and migration of atomic C species during graphene growth. The aluminum oxide barrier/Cu system has been confirmed to effectively promote the growth of graphene nanopores [105]. The size and positioning of the aluminum oxide pattern determine the corresponding features of the graphene after CVD growth. Importantly, this method circumvents the etching process, resulting in more abrupt edges with minimal damage, thereby enhancing the performance of graphene-based nanodevices produced through BG-CVD. A schematic representation of the BG-CVD process is shown in Figure 9.

The second method for bottom-up graphene nanopore manufacturing is self-assembly, which is inspired by on-surface synthesis routes for covalent carbon-based nanostructures [107]. Initially, the precursor diphenyl-10, 10′-dibromo-9, 9′-bianthracene (DP-DBBA) is dibrominated, yielding radical carbon atoms that subsequently cross-couple to form polymer chains of considerable lengths (up to 150 nm) via surface-assisted Ullmann coupling. These polymer chains are then converted into graphene nanoribbons (GNRs) through intramolecular cyclodehydrogenation. Finally, the GNRs are interconnected through H_3_ cross-coupling, resulting in the formation of the graphene nanopore structure, as shown in Figure 9c. The primary advantage of this method is that the size, density, morphology, and chemical composition of the graphene nanopores can be precisely defined at the atomic level through the design of the molecular precursors. Inspired by this strategy, the bottom-up method has been well researched for the manufacture of graphene nanopores [108,109,110,111].

#### 2.2.7. Photothermal Sculpting

**Figure 10:**
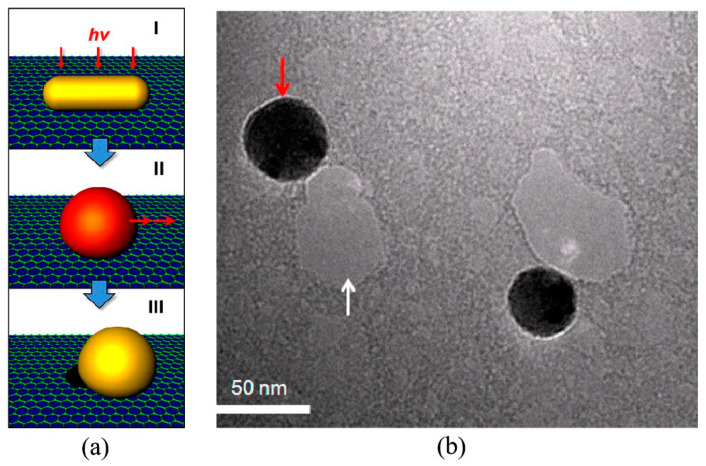
Photothermal effect drilling graphene nanopores [112]. (**a**) Illustration of photothermal sculpting process. (I) Light (hν) is illuminated on a gold nanorod on a graphene membrane, (II) the gold nanorod is melted and reshaped into a hemispherical nanoparticle (red color indicates a high surface temperature), and (III) the heated gold nanoparticle subsequently oxidizes the graphene surface to create a nanopore. (**b**) TEM image of two nanopores. Gold nanoparticles (red arrow), nanopores (white arrow).

Graphene nanopores can also be sculpted using self-integrated optical antennas through photothermal conversion [112]. This methodology is implemented through the following procedure: Initially, gold nanorods are deposited onto a freestanding graphene membrane via a drop-casting technique. Upon irradiation with a femtosecond laser, the gold nanorods exhibit pronounced photothermal conversion due to the localized surface plasmon resonance effect. During this process, the localized high temperature induced by the photothermal effect facilitates the oxidation of graphene, while the gold nanorods undergo melting and morphological reconstruction under thermodynamic forces, ultimately leading to the formation of nanopores with a diameter of approximately 50 nm on the graphene surface as illustrated in Figure 10. Research indicates that the precise regulation of laser fluence parameters and gold nanorod dimensions enables dynamic control over the morphological characteristics of graphene nanopores. It is particularly noteworthy that the selected laser wavelength must match the plasmon resonance absorption peak of the gold nanorods to ensure optimal photothermal conversion efficiency. However, it should be noted that the inherent limitations of the drop-casting technique for gold nanorod dispersion result in challenges in achieving precise control over the geometric configuration of the nanopores, with their spatial distribution exhibiting random characteristics.

## 3. Graphene Nanopore Applications

**Figure 11:**
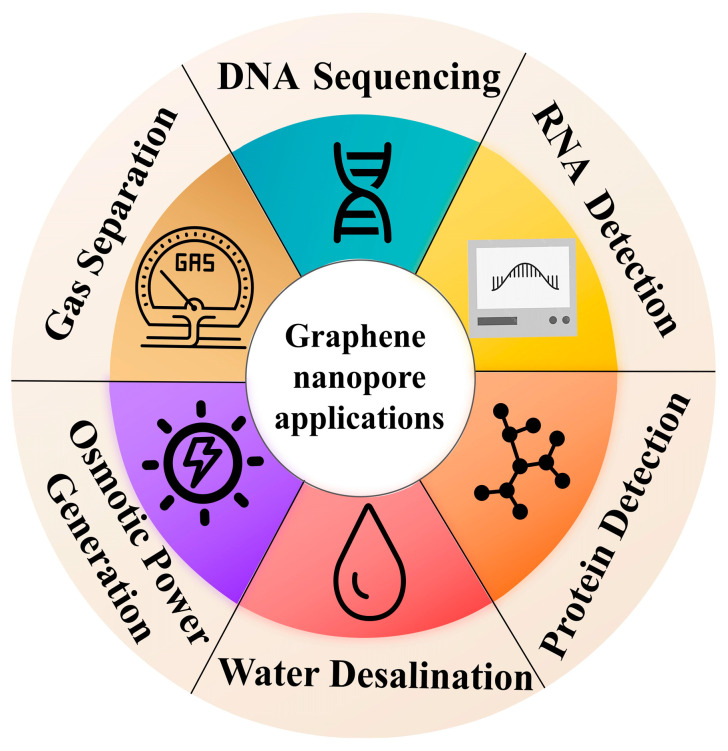
The applications of graphene nanopores.

This section comprehensively summarizes the applications of graphene nanopores, as shown in Figure 11. This discussion encompasses various areas, including biomolecule detection, water desalination, gas separation, heavy metal ion separation, and osmotic power generation. Each application highlights the unique properties of graphene nanopores and their potential for innovative solutions in these fields.

### 3.1. Biomolecule Detection

**Figure 12:**
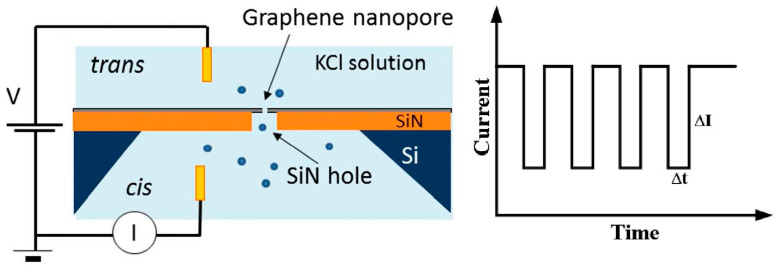
Principles of the graphene nanopore detection [115].

Due to the extremely high resolution, graphene nanopores have been widely used for biomolecule detecting, such as nucleic acids [113,114] and proteins [115]. The typical principle of graphene nanopore detection is as follows: Two cells containing an electrolyte are connected by graphene nanopores. When a voltage is applied across the nanopore, charged biomolecules are driven through it, resulting in a change in the ionic current. The magnitude (ΔI) and dwell time (Δt) of this ionic current provide valuable information for the biomolecule analysis, as shown in Figure 12.

#### 3.1.1. DNA Sequencing

The principle of the graphene nanopore sequencing method is based on the observation that each base in a DNA molecule blocks the ion current passing through the nanopore on the graphene sheet in a slightly different way. This variation occurs due to the differing sizes and shapes of the DNA molecular bases, generating different characteristic ion current signals that enable the detection of the DNA sequence. As mentioned in the introduction, graphene nanopores are recognized as sensors with the highest spatial resolution, making DNA sequencing one of their most promising applications. Over the past few decades, many scientists have investigated this application. For instance, molecular dynamics simulations have demonstrated that A-T and G-C base pairs can be discriminated using 2.4 nm graphene nanopores [116]. Subsequently, simulations by Wells et al. [117] indicated that the ionic current blockade generated by different DNA nucleotides could effectively distinguish between nucleotide types when using graphene nanopores.

**Figure 13:**
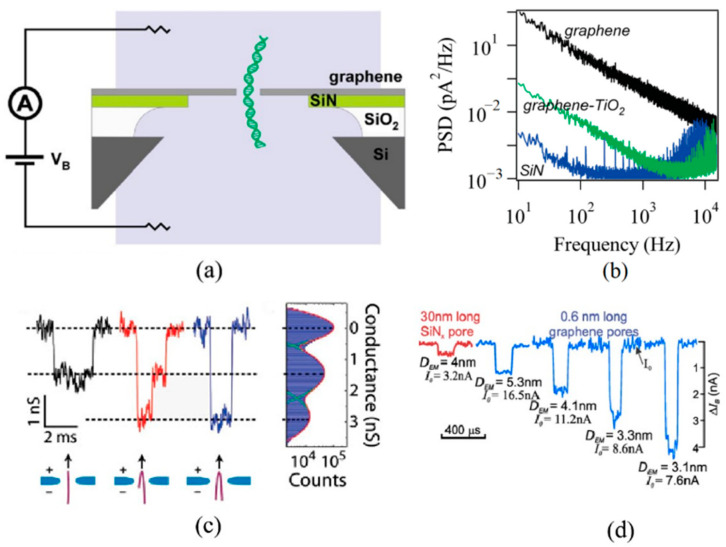
DNA translocation experiment. (**a**) Typical graphene nanopore device for DNA detecting [41]. (**b**) Power spectral density of the pore current [41]. (**c**) Different conformational signals of DNA translocation [59]. The three distinct translocation events of DNA molecules through a 22 nm pore at 200 mV correspond to: unfolded (Black), partially folded (Red), and fully folded (Blue) configurations. (**d**) Typical blockades as dsDNA translocates through Si_3_N_4_ nanopore and through graphene nanopores of different diameters [118].

In 2010, Merchant et al. [41] used electron beam lithography (EBL) to fabricate nanopores with diameters ranging from 5 to 10 nm on graphene, successfully conducting DNA translocation experiments for the first time, as shown in Figure 13a. To reduce noise, several nanometer-thick TiO_2_ layers were deposited on both sides of the graphene membrane using atomic layer deposition (ALD) [41], as shown in Figure 13b. It was also demonstrated that the blocking current of graphene nanopores was stronger than that of silicon nitride nanopores of similar pore sizes, demonstrating good sensitivity in DNA sequencing [59]. Three types of signals, unfolded, partially folded and fully folded dsDNA, were observed, as shown in Figure 13c. After that, Garaj et al. [118] compared the DNA translocation signals of Si_3_N_4_ nanopores and graphene nanopores. It was found that when nanopores from both materials were of comparable size, the blocking signal during DNA translocating through the graphene nanopores was significantly stronger, as shown in Figure 13d.

Although graphene nanopores exhibit high sensitivity for DNA sequencing, the inherent hydrophobic nature of graphene can lead to challenges during the passage of hydrophobic DNA molecules. Graphene is a two-dimensional material composed of a single layer of carbon atoms, with its surface consisting of sp^2^-hybridized carbon atoms, which exhibit a high degree of hydrophobicity. This hydrophobic nature makes the graphene surface less likely to interact with water molecules, resulting in a large contact angle of water on its surface. When DNA molecules pass through graphene nanopores, the DNA itself is also hydrophobic, particularly due to the aromatic ring structures of the nucleotide bases (such as purines and pyrimidines). These structures tend to engage in strong non-specific hydrophobic interactions with carbon atoms on the graphene surface. Such interactions cause DNA molecules to readily adsorb onto the graphene surface during their passage through the nanopore, leading to the clogging of the nanopore. To address this issue, Schneider et al. [119] developed a non-covalently bound hydrophilic coating and applied it to the surface of graphene. By designing a self-assembled monolayer (SAM) to modify the graphene surface, the hydrophobic interactions between DNA and graphene are reduced, thereby preventing nanopore clogging. Specifically, the molecule modification consists of two components: 1-aminopyrene and the N-hydroxysuccinimide ester of tetraethylene glycol monomethyl ether. The pyrene moiety of 1-aminopyrene non-covalently adsorbs onto the graphene surface through π-π stacking interactions, ensuring the firm attachment of the molecule modification, while the N-hydroxysuccinimide ester of tetraethylene glycol monomethyl ether reacts with 1-aminopyrene to form a stable amide bond, introducing hydrophilic ethylene glycol chains. These ethylene glycol chains extend into the solution, rendering the graphene surface hydrophilic and effectively preventing direct contact between DNA molecules and the graphene surface, thereby mitigating hydrophobic interactions. The modified graphene surface not only prevents DNA adsorption and nanopore clogging but also preserves the electronic properties of graphene, avoiding the alterations in the electronic structure that may result from covalent modifications. Also, mesoporous graphene hydrogel prepared by chemical etching with hydrogen peroxide first and then by an improved hydrothermal reaction has good hydrophilicity [120].

The fabricated mesoporous graphene foam (MGFP), after undergoing chemical etching and thermal annealing treatment, is further subjected to oxygen plasma treatment, thereby introducing abundant oxygen-containing functional groups (e.g., hydroxyl and carboxyl groups) on its surface, which significantly enhances the material’s hydrophilicity. This surface modification not only reduces the interfacial resistance between the electrode and the electrolyte but also ensures that all pore channels actively participate in the capacitive deionization process, ultimately improving the ion adsorption performance of the material. Recently, Zhou et al. [121] successfully used graphene nanopores to distinguish between translocation signals of two distinct DNA homopolymers, underscoring the significant potential of graphene nanopores in DNA sequencing applications.

Theoretical studies have demonstrated that a graphene nanoribbon containing nanopores can effectively identify all four DNA bases with an enhanced bandwidth [122,123,124]. As DNA translocates through the nanopore, specific non-electrostatic interactions between the DNA molecules and the graphene nanoribbon alter the local density of states around the nanopore. This interaction leads to a change in the resistance of the graphene nanoribbon, inducing a variation in the current flowing across its surface. Consequently, DNA bases can be identified by monitoring these changes in the in-plane current. This is because the current through the graphene nanoribbon is significantly higher than the ionic currents within the nanopore, providing a wider bandwidth for DNA detection [123].

**Figure 14:**
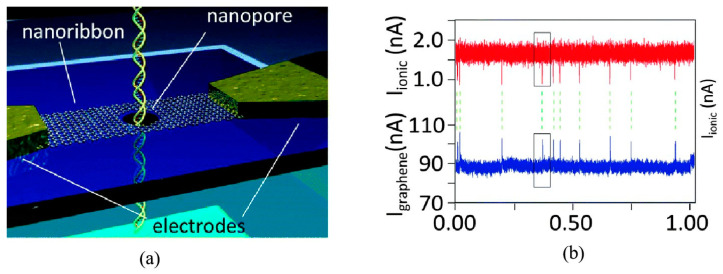
A graphene nanoribbon for detecting DNA [47]. (**a**) A schematic diagram of a biomolecule passing through graphene nanopores. (**b**) Simultaneously recorded ionic current and electrical current flowing through the GNR during translocations of pNEB DNA in 10 mM KCl (transmembrane voltage, 200 mV; graphene source–drain voltage, 20 mV). Ionic current is displayed in red and graphene current in blue.

In 2013, Travers et al. [47] were the first to successfully detect DNA translocation through graphene nanoribbons with a nanopore, as shown in Figure 14a. The current through the graphene nanoribbon correlates with the occurrence of blocking currents, as shown in Figure 14b. Recently, a differential current amplifier was developed, which can distinguish capacitive current signals and resistive response signals on nanoribbons effectively [125]. Capacitive currents emerge from temporal fluctuations in potential, coupled with the capacitance existing between the electrolyte and the conducting channel of the sensor. When conducting DNA translocation experiments with graphene nanostructure devices, it was necessary to simultaneously record both the ionic nanopore current and the graphene transverse current. This endeavor posed significant challenges, stemming from the intricate nature of the fabrication process and the difficulties associated with effectively wetting the nanopores. The inherent hydrophobic properties of graphene likely exacerbated the latter challenge. Notably, the samples could not undergo treatment with oxygen plasma or piranha solution, as these methods would degrade the graphene structure. Attempts to wet the pores through the use of ethanol flushing frequently led to structural damage, drastically diminishing the success rate of the experiments. Despite these formidable obstacles, we were able to secure consistent data from a single sample (out of the initial 180 devices fabricated), which exhibited a sufficient signal-to-noise ratio for a thorough analysis. Due to the substantial magnitude of the in-plane current in the nanoribbon, the in-plane current detection method demonstrates a stable frequency response at high sampling frequencies (>MHz). This enables the differential current amplifier to sample at high translocation velocities. However, the sophisticated design of graphene nanoribbon–nanopore devices presents considerable challenges in the manufacturing process.

Graphene nanopore sequencing is a promising sequencing method that has demonstrated excellent spatial resolution in DNA detection [50]. However, several challenges remain, including the need for the mass production of high-quality graphene nanopores at a low cost, the development of improved detection methods, and the control of DNA translocation speed to achieve high-resolution detection. Additionally, the low signal-to-noise ratio of graphene complicates the differentiation of the four nucleobases.

#### 3.1.2. RNA Detection

RNA sequencing provides critical insights for early disease diagnosis and prognostic assessment [126,127], including information on biological transcription status, the types of bacteria and viruses, and gene expression levels. For instance, Wanunu et al. [32] used a graphene nanopore sensor to detect miRNA extracted from histocytes, while Wang et al. [128] used nanopores to identify microRNAs in lung cancer patients, using oligonucleotide probes for selective detection in plasma. Graphene nanopore detection shows good sensitivity, capable of detecting picomolar concentrations in a direct and label-free manner. Consequently, these nanopores are powerful tools for quantitatively studying microRNA and identifying disease markers, holding significant potential for early disease diagnosis.

Theoretically, graphene nanopores represent the highest-resolution nanopore sensors, playing a crucial role in RNA detection. Molecular dynamics (MD) simulations [129] have shown that nanopore technology can capture the electrical signal changes produced during the translocation of a single RNA molecule through a nanopore, enabling the accurate identification and quantitation of microRNAs. This technique not only allows for the detection of microRNAs at low concentrations but also provides detailed sequence information, which is vital for understanding their roles in the onset and progression of lung cancer. By comparing the results across different pore sizes, Onanuga K et al. found that graphene nanopores with diameters less than 3 nm can effectively identify modify tRNA. Furthermore, graphene nanopores offer several advantages such as efficient selective separation, high mechanical strength, excellent water permeability, and environmental sustainability in seawater desalination. Therefore, we believe that graphene nanopores hold significant value in the field of RNA detection.

#### 3.1.3. Protein Detection

Protein sequencing reveals the biological mechanism of life activities and disease development [130]. Nanopore detection technology enables the real-time, single-molecule detection of proteins, thus exhibiting extremely high sensitivity and resolution. This capability facilitates the precise identification and analysis of protein structures and functions. In comparison to DNA, proteins exhibit an uneven charge distribution, the presence of hydrophobic domains, a relatively low surface charge density, and more complex structures, which complicate protein detection [50].

Shan et al. [131] detected ferritin using graphene nanopores. To prevent ferritin adsorption on the graphene surface, they employed oxygen plasma to modify the surface; however, protein adsorption still occurred. Schneider et al. [119] developed a general strategy to non-covalently modify the hydrophobic surface of graphene by designing a dedicated self-assembled monolayer of pyrene ethylene glycol, rendering the surface hydrophilic. This modification prevents DNA adsorption on graphene and enables the detection of single-stranded DNA in graphene nanopores with enhanced durability and reproducibility. Furthermore, the heterojunction nanopores fabricated by combining graphene with MoS_2_ thin films effectively mitigate the adhesion of protein molecules during translocation through the nanopores while extending the translocation time [132]. This is primarily attributed to the distinct van der Waals interactions between protein molecules and these two materials. As a result, protein molecules tend to migrate from the MoS_2_ surface to the graphene surface, where they become stagnant. Notably, utilizing this heterojunction nanopore configuration, the successful detection of bovine serum albumin (BSA) molecule translocation signals has been achieved. Goyal et al. [133] also distinguished interaction signals of immunoglobulin (IgG) antibodies using graphene nanopores, demonstrating their utility in detecting single-protein molecules and protein–protein interactions. MD simulations have shown that graphene nanopores can distinguish different subgroups of proteins, such as IgG_2_ and IgG_3_, based on variations in translocation time and ion current [134].

Theoretical and experimental studies show that graphene nanopores have achieved single-based detection. However, despite this significant advancement, several challenges hinder the practical application of this technology. One of the primary concerns is signal noise, which poses a major concern in single-molecule detection. When detecting individual molecules, graphene nanopores can be affected by interference from the surrounding environment, such as the influence of other molecules in solution or thermal noise, leading to signal instability and complicating accurate identification. Furthermore, the interactions between biological molecules, like RNA or DNA, and the graphene nanopores can affect detection results. Factors such as the orientation, speed and conformation of the molecules passing through the nanopores may also influence the signal. Nonetheless, graphene nanopores remain a promising sensor for biomolecule detection with high accuracy.

### 3.2. Material Separation

The permeance of separation membranes is inversely proportional to their thickness, making it a challenge to reduce the thickness of separation membrane materials to improve separation efficiency. According to Fick’s First Law, the steady-state diffusion flux *J* is directly proportional to the concentration gradient Δ*C* and the diffusion coefficient *D* while inversely proportional to the mass transfer path length (i.e., membrane thickness *L*), expressed as *J*∝*D*Δ*C*/*L*. In pressure-driven separation processes such as reverse osmosis, the solution–diffusion model further reveals that the water permeability coefficient *A* is inversely proportional to the membrane thickness (*A*∝*DwSw*/*L*, where *Dw* is the diffusion coefficient and *Sw* is the solubility coefficient), indicating that reducing membrane thickness significantly enhances permeation flux. Experimental studies have confirmed that single-layer graphene nanopore membranes (thickness ≈ 0.34 nm) exhibit water permeance 2–3 orders of magnitude higher than conventional polyamide membranes [135], while the permeability of graphene oxide (GO) membranes increases exponentially with a decreasing layer number [136]. However, the fabrication of ultrathin membranes necessitates a trade-off between mechanical strength and selectivity: although reduced thickness lowers mass transfer resistance, structural defects or uncontrolled pore sizes may compromise selectivity. Consequently, the design of ideal separation membranes requires the synergistic optimization of thickness, pore size distribution, and surface chemistry, alongside stability-enhancing strategies such as support layers or chemical crosslinking [137]. A single-atom-thick graphene nanopore membrane represents an ideal membrane for material separation, such as desalination, heavy metal ion separation and gas separation. The π-orbital of graphene forms dense, delocalized electron clouds [138] that fill the gaps within its aromatic rings, generating a repulsive field that can prevent molecules from passing through [139]. This repulsive field arises from electronic interactions within the material; as the π-orbitals overlap and electrons move freely, they create a repulsive force that helps maintain the structural integrity of the graphene lattice. Consequently, this repulsive field can effectively block the transmission of specific molecules based on their size, shape, and chemical properties.

#### 3.2.1. Water Desalination

**Figure 15:**
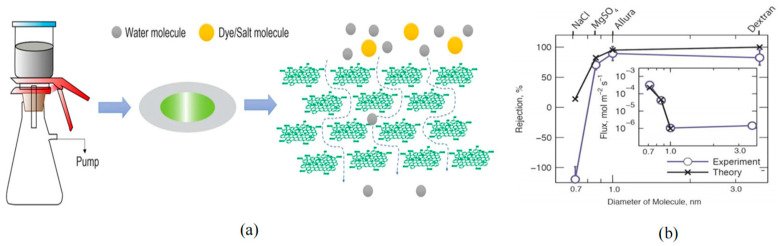
Graphene nanopores for water desalination. (**a**) The principle of graphene nanopores for water desalination [146]: the nanopore allows water molecules across the graphene membrane (right), while salt ions (spheres) are blocked. (**b**) Graphene nanopore’s ability to reject four solutes (NaCl, MgSO_4_, Allura Red and dextran) [142].

Water desalination is one of the most promising solutions to address the shortage of freshwater resources. However, existing desalination technologies often require large energy consumption and high costs. Graphene nanopores have emerged as a potential solution to these challenges. For seawater desalination, the size of graphene nanopores needs to be less than 0.45 nm to allow water molecules to pass through while retaining other molecules and ions, as shown in Figure 15. Cohen-Tanugi et al. [135] were the first to propose the concept of using graphene nanopores for water desalination. They extensively researched the relationship between desalination performance, pore size, chemical functional groups and applied pressure. The results show that most salt ions can pass through pores with a diameter greater than 0.55 nm. Additionally, the chemical functional groups present on the nanopore surface significantly affect desalination ability and water permeability. For a given pore size and applied pressure, hydroxylated pores exhibit a lower desalination rate compared to hydrogenated pores. The hydrophilic nature of hydroxyl functional groups tends to hinder water transport and interact with salt ions, leading to reduced desalination efficiency. In contrast, hydrogenated pores have simpler surfaces and weaker interactions with water molecules and salt ions, facilitating smoother water transport and lower salt ion permeability, which results in a higher desalination rate. Konatham et al. [140] found that effective ion repulsion can be achieved using a non-functional pore with a diameter of about 0.75 nm. Theoretical research has also shown that the desalination performance of graphene nanopore membranes surpasses that of traditional polymer filtration membranes.

In pursuit of greater seawater desalination efficiency, Surwade et al. [92] prepared graphene nanopore arrays using oxygen plasma etching. Graphene nanopores exhibited an excellent water penetration rate (10^6^ gm^−2^ s^−1^) and efficient desalination rate (close to 100%). In addition, other studies have found the rejection of graphene nanopores towards four different molecules: NaCl, MgSO_4_, Allura Red, and dextran. As shown in Figure 15b, the larger the molecular size, the higher the rejection rate of graphene nanopores. Subsequently, Gupta S et al. [141] introduced two types of graphene membranes for seawater desalination: shear-aligned graphene oxide (SA GO) membranes and porous graphene (HG) membranes. By applying shear force, the liquid crystalline phase of graphene oxide aligns to form ordered nanochannels. These membranes have a thickness of approximately 140 ± 20 nm. The SA GO membranes exhibit high water permeability (approximately 70 ± 12 L m^−2^ h^−1^ bar^−1^) and an efficient retention of organic molecules (about 95%) while also significantly removing monovalent and divalent ionic salts (with removal rates ranging from 40% to 70%). With catalytic metal oxidation, the pore size distribution can be precisely controlled, resulting in porous graphene membranes with a narrow pore size distribution. The HG membranes not only possess exceptional water permeability but also demonstrate extremely high ion selectivity, particularly in the removal of monovalent and divalent ions. Additionally, the study explored the potential of utilizing xylem plants (such as white pine) as natural filtration media. The conduit structures of these plants resemble nanoporous membranes, enabling the effective filtration of organic dyes from water. Furthermore, scholars have investigated the technology of nanofiltration using monolayer nanoporous graphene membranes with defects sealed, demonstrating its feasibility in filtration at the ionic and molecular levels. They have also discussed the potential applications of this technology in water resource purification and reuse, thereby providing a platform for research on nanofluidic transport in membranes made of graphene and other ultrathin materials [142].

Kazemi et al. [143] fabricated a large-area graphene nanopore array of 2.77 × 104 μm^2^ on a graphene membrane supported by TEM grids, achieving a salt rejection rate of 76%. However, the pore size necessary for effective water desalination via size exclusion is under 5.6 nm, which may limit the desalination flux. By modifying the surface charge of graphene nanopores, electrostatic repulsion can enhance the desalination ability of larger pore sizes. Furthermore, Li et al. [144] studied the repulsion of Cl^−^ ions in negatively charged graphene through molecular dynamics simulation. They found that increasing the surface charge density effectively prevents Cl^−^ from passing through the nanopore. However, if the surface charge density exceeds the critical value, more ions may flow into the nanopore to maintain charge neutrality, thus reducing desalination efficiency. Their simulations indicated that a graphene nanopore with a diameter of 3.5 nm exhibits excellent Cl^−^ ion exclusion capabilities at surface charge densities ranging from −0.09 C/m^2^ to −0.12 C/m^2^. The combination of positively and negatively charged graphene pores facilitates the removal of both cations and anions, achieving efficient water desalination. Recently, the integration of graphene with other two-dimensional materials provided new ideas for graphene nanopores in the field of seawater desalination. Guo et al. [145] demonstrated water desalination through the electric field-induced ion concentration depletion effect on the 2D nanofluidic heterojunction. Li et al. [144] also explored the potential of graphene–MoS_2_ heterostructure membranes for seawater desalination, with a focus on bilayer membranes and their advantages over single-layer membranes. Through extensive molecular dynamics simulations and statistical analyses, the graphene–MoS_2_ bilayer membrane was studied and compared with the single-layer membranes of graphene and MoS_2_, respectively. It was found that the bilayer graphene–MoS_2_ heterostructure membrane achieved improved water flux while maintaining a high ion rejection rate.

In addition to filtering Na^+^ and Cl^−^ ions in seawater desalination, graphene nanopores are also effective for the removal of other heavy metal ions, such as Zn^2+^, Fe^3+^, Fe^2+^, and Cu^2+^. By controlling the pore size and functional groups on the graphene surface, the selective rejection of metal ions can be achieved. Using this property, graphene nanopores can effectively remove metal ions. Azamat et al. [147] employed MD simulations to investigate the removal of Zn^2+^ using graphene nanopores. Their research found that only Zn^2+^ permeates through functionalized graphene nanopores when a certain voltage threshold is applied, while Cl^−^ are blocked. The reason may be that the energy barrier for Cl^−^ is higher than that of Zn^2+^ in graphene nanopore membranes. Based on this research, Kommu et al. [148] explored the removal of mental ions by graphene nanopores functionalized with various groups, including hydroxyls, nitrogen, and fluorine. Their research showed that nitrogen-functionalized graphene nanopores exhibit the highest permeability. Zhan et al. [149] fabricated graphene nanopores of PET substrates using swift heavy ion irradiation and conducted Fe^3+^/Fe^2+^ separation experiments. They proposed that ion permeation occurs through ion exchange between metal ions and protons on either side of graphene nanopores, and the electric field can increase the permeability of ions through the membrane, but this will reduce the separation performance of the graphene nanopores. Similarly, Li et al. [150] performed simulations to evaluate the separation efficiency of Cu^2+^ using graphene nanopores functionalized with hydroxyl, nitrogen, and boron. Their results indicated that boron-functionalized graphene nanopores exhibit three times the permeability of untreated graphene nanopores. However, due to ion accumulation at the pore interface, the ion concentration increases, leading to a significant reduction in membrane permeability over time.

Recently, evaporation-based solutions were seen to offer the advantage of low energy consumption, and a 10−30-fold enhancement in water evaporation flux was observed through Å-scale graphene nanopores [151]. Ronghe A et al. [152] conducted molecular dynamics simulations to investigate the suitability of Å-scale graphene nanopores in enhancing water evaporation from salt solutions (LiCl, NaCl, and KCl). The study revealed that cation–π interactions between ions and the surface of nanoporous graphene significantly influence the ion populations in the vicinity of the nanopores, leading to varied water evaporation fluxes among different salt solutions. Notably, the highest water evaporation flux was observed in KCl solutions, followed by NaCl and LiCl solutions, with these differences diminishing at lower concentrations. Compared to the bare liquid–vapor interface, 4.54 Å nanopores exhibited the most pronounced enhancement in evaporation flux, ranging from 7- to 11-fold. Notably, an enhancement of 10.8 was achieved for a 0.6 M NaCl solution, which closely mimics the composition of seawater.

In conclusion, atomically thin graphene nanopores can significantly enhance permeability, while their good mechanical properties allow them to withstand higher pressure, thereby increasing water flow rates. Conventional reverse osmosis (RO) membranes rely on high pressure (approximately 5–7 MPa) to drive seawater through dense polymer membranes, with energy consumption ranging from 1.8 to 5 kWh/m^3^. The high permeability of graphene membranes implies a significant increase in water production per unit area under the same pressure, potentially achieving equivalent water output at lower pressures, thereby substantially reducing energy consumption. Furthermore, due to the low permeability of traditional RO membranes, large membrane areas and high-pressure systems are required to meet demand. The high permeability of graphene membranes can reduce equipment size and lower infrastructure costs, making them particularly suitable for remote areas or mobile water treatment units. Additionally, the faster water production rates can accelerate seawater desalination or wastewater treatment, alleviating pressure on freshwater scarcity. Theoretically, graphene nanopores can purify water at a rate of 10–100 L/cm^2^/MPa/day, which is 2–3 orders of magnitude higher than RO membranes [135]. On the other hand, the precise control of pore size and functional group modifications can enhance ion selectivity, thereby improving desalination performance. Graphene nanopores has shown great potential in the field of seawater desalination and the industrial wastewater treatment of heavy metal pollution. However, current challenges hindering their widespread application include the difficulty in producing defect-free graphene sheets and transferring them onto substrate materials, as well as the need for the rapid, efficient, and precise fabrication of nanopore arrays with appropriate pore diameters.

#### 3.2.2. Gas Separation

Another potential application of graphene nanopores is as a gas separation membrane. By creating suitably sized nanopores on graphene, small molecules are allowed to pass through, and large molecules are prevented from passing through. Jiang et al. [52] proposed that graphene nanopores can be used to separate methane (CH_4_) and hydrogen (H_2_). The theoretical calculations results show that H_2_ can pass through the pores with a 0.25 nm diameter, while CH_4_ is difficult to pass through. Since then, a large number of theoretical studies have proved the ability of graphene nanopores to separate gasses such as CO_2_/CH_4_, N_2_/CH_4_, CO_2_/N_2_, and H_2_/N_2_ [153,154,155,156]. Sun et al. [157] used MD to simulate the ability of graphene nanopores to separate CO_2_/CH_4_ mixtures. They found that negatively charged graphene nanopore membranes can improve the separation performance of CO_2_/CH_4_ mixtures. For example, when the membrane charge changes from neutral to −0.125 e, the CO_2_ permeability increases, and the selectivity increases from 10.4 to 42.8. Later, based on this research, they also simulated and analyzed the separation of CO_2_/N_2_ by graphene nanopores and drew a similar conclusion [158]. Unfortunately, there is a lack of experimental evidence for the gas separation capability of graphene nanopores, which may be attributed to the fact that there are still some issues in the manufacturing process of graphene nanopores.

**Figure 16:**
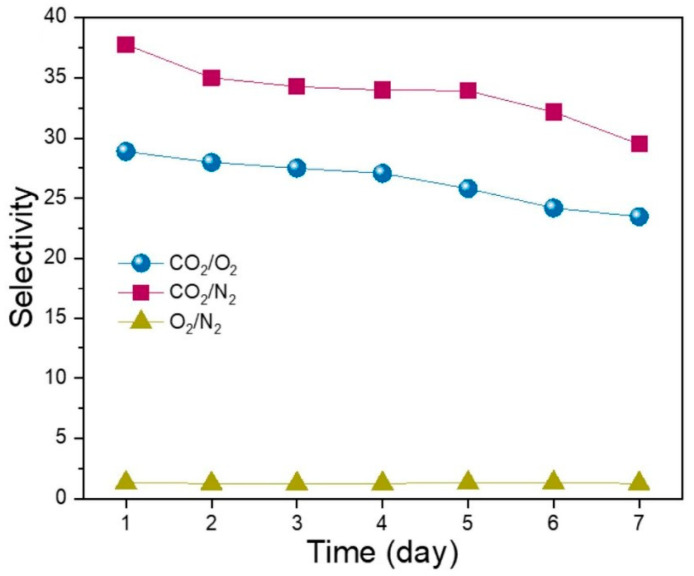
Gas leak rates of graphene nanopore membrane [139].

Koenig et al. [139] used graphene nanopores to measure the transport of various gasses (H_2_, CO_2_, Ar, N_2_, CH_4_ and SF_6_); the experimental results showed that the molecular selectivity and leak rates decreased with the increase in molecular sizes, as shown in Figure 16. After that, Zhao et al. [94] completed the H_2_/CH_4_ separation experiment by high-density graphene nanopores. The graphene nanopore membrane showed a high separation performance (the H_2_/CH_4_ separation factor is 15.6 to 25.1). Recently, scholars have proposed embedding crown ethers into graphene nanopores to enhance the efficiency of gas separation [159]. Employing MD simulation methods, they investigated the separation performance of three crown ether nanopores for CO_2_/CH_4_ and CO_2_/CO gas mixtures. The simulation results reveal significant improvements in gas separation efficiency achieved by the graphene nanopores embedded with crown ethers, offering a novel perspective for future gas separation processes. This study underscores the potential of such hybrid nanostructures in advancing gas separation technologies. Until 2018, the size screening behavior of nanopores in monolayer graphene was proved by directly measuring the composition of mixed gasses passing through nanopores [160]. In addition, scholars have enhanced the sensitivity to carbon monoxide gas by combining non-hexagonal symmetric T-shaped graphene with lithium (Li) atoms. These research findings provide theoretical and experimental guidance for the development of novel gas sensors based on T-shaped graphene [161].

Gas separation technology plays an important role in industry and scientific research, and graphene nanopore membranes are emerging as ideal materials for gas separation. Their atomically thin structure enhances permeability, while their molecular selectivity can be adjusted to separate different gasses by controlling the pore size. Numerous simulations have assessed the potential of graphene nanopores for gas separation; however, further experimental research is necessary to validate these theoretical findings. We believe that graphene nanopores will play an increasingly significant role in gas purification, pollution prevention, greenhouse effect management, and so on.

### 3.3. Osmotic Power Generation

**Figure 17:**
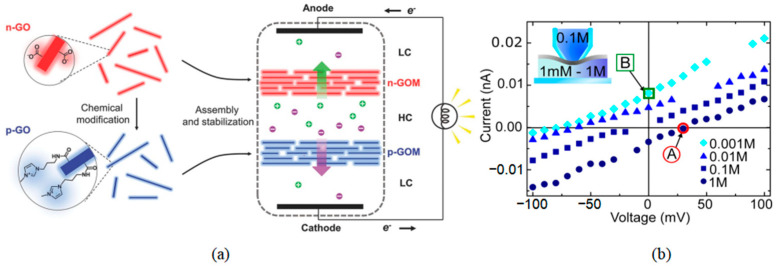
Graphene nanopores for osmotic pressure power generation. (**a**) The principle of osmotic power generation [163]. (**b**) Typical I–V curves for graphene nanopore membrane. Ion diffusion causes a positive current which is indicated by the green square (B) [166]. The voltage to stop this current is indicated by red circles (A).

Using the osmotic pressure difference between freshwater and seawater to generate electricity is a renewable and environmentally friendly method. In this method, different concentrations of electrolytes are separated by a thin membrane with nanopores, and the osmotic potential generated by the pressure gradient or salt concentration gradient is used to drive the electrolyte through the nanopore, thus generating an osmotic current [162,163], as shown in Figure 17a. Since the water permeability through a membrane is inversely proportional to its thickness, graphene exhibits significant advantages in osmotic power generation. Gai et al. [164] used MD to simulate the water transport process in graphene nanopores. The simulation results show that the water flux through graphene nanopores is three times that of a typical cellulose membrane, with a correspondingly higher power density. Similarly, research on osmotic energy conversion through ultrathin nanopores has revealed significant insights into the origin of a diffusion current and membrane potential [165]. A systematic investigation demonstrated that ultrathin nanopores with a pore size of 1–2 nm and a porosity of 10% can achieve a remarkable power density approaching 200 Wm^−2^. These findings indicate that such nanoporous structures exhibit exceptional potential for large-scale osmotic energy conversion applications. The study further elucidated the fundamental mechanisms underlying the energy conversion process in nanoconfined systems, providing valuable guidance for the development of high-performance osmotic power generation systems. Walker et al. [166] carried out concentration-driven diffusion experiments of cations and anions through graphene nanopores. When a concentration difference of 100 times was applied, a significant diffusion current could be detected, as shown in Figure 17b. They calculated that the power generated by graphene’s osmotic pressure can reach 700 Wm^−2^, far exceeding the performance of traditional membranes (0.1–10 Wm^−2^) [164,167,168,169]. Recent investigations into the transport phenomena of simple liquids through nanoporous graphene membranes have yielded significant insights. A comprehensive study employing Non-Equilibrium Molecular Dynamics (NEMD) simulations systematically examined the influence of pore size variations on liquid transport characteristics [170]. The results demonstrated a distinct correlation between pore dimensions and flow dynamics, revealing that reduced pore sizes lead to enhanced velocity profiles, particularly at both the pore center and the liquid–solid interface. This observed behavior was mechanistically explained by the intensified van der Waals interactions occurring between the confined liquid molecules and the graphene wall atoms. These findings provide a fundamental understanding of nanoscale fluid transport mechanisms, which is crucial for advancing the development of porous graphene-based filtration and separation technologies. Nevertheless, due to the dominance of van der Waals repulsive forces at the molecular level, the slip velocity, interfacial viscosity, and the influence of pore boundaries exhibited an exponential decay as the pore diameter increased. Recently, it was proved that the introduction of in-plane nanopores on GO sheets can inhibit structural defects in the membrane (such as wrinkles, voids, and folded layers). When used as a nanofluidic membrane in the osmotic power generation system, the porous GO membrane exhibits a higher osmotic power density (13.15 Wm^−2^) and conversion efficiency (46.6%) than the original GO membrane under a 1000-fold KCl concentration gradient [171].

Graphene nanopores are promising membranes for osmotic power generation. On the one hand, their single-atom thickness ensures high ion permeation efficiency, leading to greater power density. Additionally, their good mechanical properties allow them to withstand high driving pressures without damage [172]. Although graphene nanopore power generation remains in the experimental research stage, we believe that with the maturity of graphene nanopore manufacturing technology in the future, graphene nanopores could play an important role in the development of blue energy.

## 4. Conclusions

As summarized above, due to their high spatial resolution and ion selectivity, atomic thinness, and ion selectivity following pore fabrication, graphene nanopores have attracted significant attention and undergone rapid advancement in recent years. This review highlights both the fabrication methods and applications of graphene nanopores. Among the fabrication methods mentioned in this article for graphene nanopores, plasma etching is the most valuable approach. It offers a cost-effective method for producing graphene nanopores and benefits from the controllability provided by template-assisted etching. This technique enables the rapid generation of large-area graphene nanopores with periodic arrays. Both theoretical and experimental studies have demonstrated the capability of graphene nanopores to achieve single-based detection, which is critical for understanding the fundamental building blocks of biological genetic information and the pathogenesis of diseases. So far, a 4-inch graphene membrane can be fabricated by the CVD method [173,174]. This wafer-level fabrication technology has made it possible for graphene nanopores to be applied in various fields widely.

However, several challenges must be addressed before the commercial application of graphene nanopores becomes feasible. First, the size of the nanopores must be precisely controlled to achieve optimal sensitivity, a task that current fabrication techniques struggle to accomplish with the required precision. Second, the hydrophobic interaction between graphene and biomolecules like DNA or RNA can cause the DNA to adsorb onto the graphene surface, obstructing the nanopores [119]. In addition, graphene nanopores have high 1/f noise, which can compromise their performance. To address the issues of DNA molecule adhesion to graphene nanopores and high noise levels, Schneider et al. [119] developed a specialized self-assembled monolayer. This hydrophilic coating, consisting of a combination of a hydrophobic aminopyrene molecule and a hydrophilic N-hydroxysuccinimide derivative of a 4-mer ethylene glycol molecule, effectively prevents DNA adsorption near the nanopores. Additionally, noise levels can be reduced by depositing TiO_2_ on the graphene surface [41], although this method sacrifices spatial resolution. Compared with DNA, proteins have an uneven charge distribution, the presence of hydrophobic domains and relatively low surface charge density, and more complex structures, meaning that protein detection is more difficult [50].

Finally, the preparation efficiency of graphene nanopores is still relatively low, making it difficult to meet the demands of large-scale production and applications. It is noteworthy that graphene is inherently imperfect, featuring inevitable intrinsic defects on its surface, which implies that the preparation process of graphene nanopores and related materials inevitably introduces defects. Clarifying the potential mechanisms of these defects and the potential impacts of the corresponding defect structures represent crucial aspects that deserve attention in future research endeavors. For instance, recent studies on the BN counterpart of biphenylene networks and the giant piezoelectricity in B/N-doped 4, 12, 2-graphyne have provided valuable insights into defect engineering and the modulation of material properties [175,176].

Additionally, the choice of preparation methods and conditions significantly influences the defect structures. Consequently, exploring a cost-effective, large-scale, and controllable defect preparation technology poses the most formidable challenge for the industrial application of graphene nanopores [177]. Additionally, energy consumption and material waste during the fabrication process remain challenges that need to be addressed. In applications such as material separation and osmotic pressure power generation, the precise control of the size distribution in graphene nanopore arrays is crucial for selectively allowing specific molecules to pass through while blocking others.

Currently, achieving the low-cost, scalable, and controllable manufacturing of graphene nanopore arrays continues to pose significant difficulties. In summary, with the advancement of micro/nano manufacturing technologies, the development of controllable and cost-effective methods for fabricating graphene nanopores is expected to be realized, enabling their broader application across various fields.
